# Combined posterior flap and anterior suspended flap dacryocystorhinostomy: A modification of external dacryocystorhinostomy

**DOI:** 10.4103/0974-620X.60016

**Published:** 2010

**Authors:** Amarendra Deka, S. P. Saikia, S. K. Bhuyan

**Affiliations:** Bawri Nethralaya, Shillong, Guwahati, India; 1 Regional Institute of Ophthalmology, Shillong, Guwahati, India

**Keywords:** Chronic dacryocystitis, epiphora, external dacryocystorhinostomy, lacrimal, success rate

## Abstract

**Background:**

External dacryocystorhinostomy (DCR) remains a reliable surgical technique for the treatment of obstruction of lacrimal drainage system beyond the common canalicular opening.

**Aim:**

To describe a simple modified double flap external DCR technique.

**Materials and Methods:**

Ninety six consecutive cases of chronic dacryocystitis with or without mucocele were selected irrespective of age and sex. In a modification to routine external DCR, a modified technique was followed, where both anterior and posterior flaps of lacrimal sac and nasal mucosa are created and sutured. Two double armed sutures were used to join the edges of anterior flaps, and elevate them anteriorly to avoid adhesion or apposition with underlying sutured posterior flaps, and to approximate the deep plane of the wound.

**Results:**

At the end of average follow-up period of 13 months, we observed 98.9% objective and 96.8% subjective success rates. The average operation time was 45 minutes. No significant intraoperative or postoperative complications were noticed.

**Conclusion:**

We believe that combined posterior flap and anterior suspended flap DCR technique is simple to perform and has the advantage of both double flap DCR and anterior suspension of anterior flaps. The results of the study showed the efficacy of this simple modification.

## Introduction

Epiphora or abnormal tearing occurs because of blockage in the lacrimal drainage system. External dacryocystorhinostomy (DCR) that involves fistulization of lacrimal sac into the nasal cavity may alleviate the symptoms. Toti (1904, Italy) first described the technique of external DCR.[1] Dupuy-Dutemps and Baerrget (1921, France) described the modern external flap DCR technique.[2] Since then, DCR has proved to be a reliable operation for obstruction beyond the common canalicular opening. Though double flap DCR is the toughest of anastomoses, it produces excellent results in skilled, experienced hands.[3]

Numerous modifications in various surgical steps of the original DCR procedure have been introduced over the years for a better surgical out come without altering its basic concept. In this prospective study, we share our experience of a new modification in external DCR technique. This modified technique of external DCR has not been published previously to the authors’ knowledge. The aim of the study was to describe the efficacy in determination of intra and postoperative complications, as well as the success rate of a new modification of external DCR technique, combined posterior flap and anterior suspended flap DCR.

## Materials and Methods

This prospective interventional study was conduced from June 2005 to July 2008. Ninety six consecutive cases of chronic dacryocystitis with or without mucocele were selected, irrespective of age and sex. A complete history was taken in every case and thorough clinical examinations were performed. Preoperatively, irrigation and probing was performed. Tear meniscus height was measured in each case. External lacrimal fistula in chronic dacryocystitis cases and failed DCR cases were excluded. The ethic review board of Bawri Nethralaya, Meghalaya approved this study.

### Surgical technique

All operations were conducted under local anesthesia. With the patients under anesthesia, the nasal cavity of the operable side was decongested for 10 minutes with cotton pledgets soaked in 2% lidocaine with adrenaline (1:200000) and 0.025% xylomethazoline.

A curved 11 mm skin incision was placed 3.5 mm nasal to the medial canthus. Next the orbicularis muscle was bluntly dissected and anterior limb of the medial canthal tendon and periostium were exposed. The skin and the orbicularis muscle were then raised medially and laterally with two cats paw forceps. The exposed periostium was incised parallel to the anterior lacrimal crest and an osteotomy of 12 × 12 mm wide was created with the Citelli’s bone punch.

**Figure 1:**
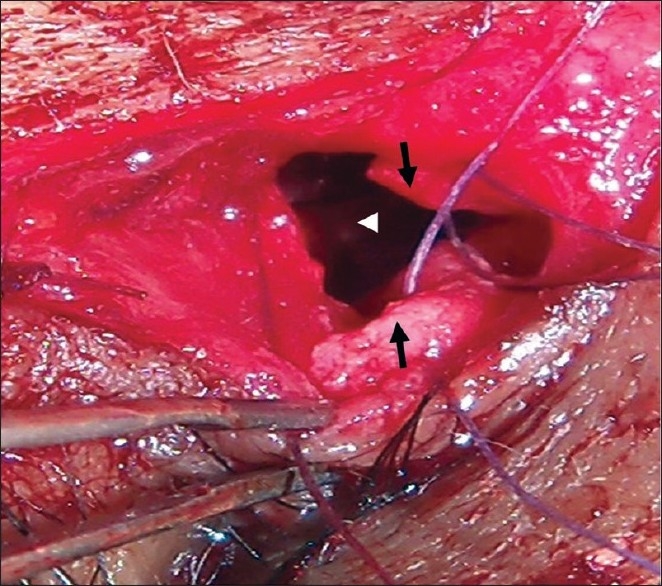
Anterior flaps of lacrimal sac and nasal mucosa (arrows) being sutured with two double arm 6-0 vicryl sutures after suturing the posterior flaps (arrowhead)

**Figure 2:**
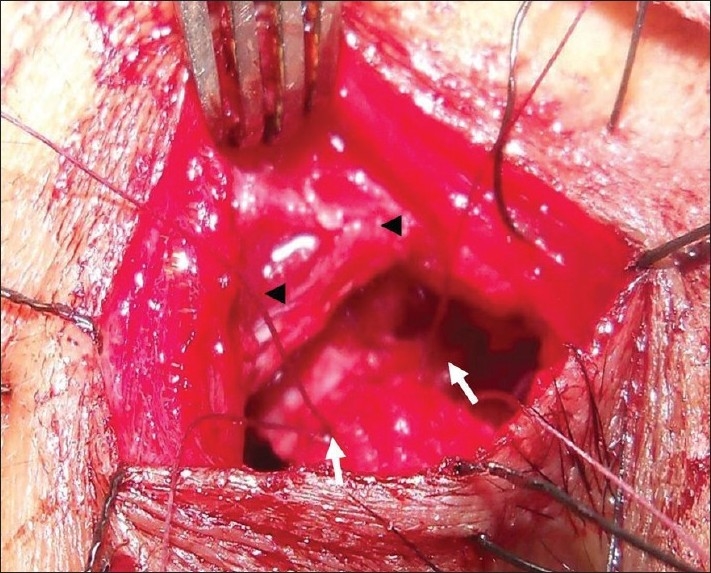
Both the anterior flaps are sutured at superior and inferior edges of the flaps (arrows). The sutures (stars) are then passed through the orbicularis muscle (arrowheads) and tied to close the deep plane of the wound

Then, anterior and posterior flaps of lacrimal sac and exposed nasal mucosa in the middle meatus were created. The posterior flaps of the lacrimal sac and nasal mucosa were sutured with 6-0 vicryl suture at the two ends [Figure 1]. Next, two double arm 6-0 vicryl sutures were passed through the superior and inferior corners of the two anterior flaps of sac and nasal mucosa and knots were tied but the sutures were not cut [Figure 2]. In the 2^nd^ step, one arm on one side of each knot is passed through the orbicularis oculi at the subcutaneous level on one edge of incision. The other arm of each knot is again passed through the orbicularis oculi at the subcutaneous level in the other edge of the incision. Then, in the third step, the upper and lower arms are tied separately making two knots. By this modification, we joined the two anterior flaps, as well elevated them forward eliminating the possibility of adhesion with underlining tissues. At the same time, deep planes of the wound (orbicularis oculi) were approximated. The skin wound was closed with continuous 6-0 vicryl sutures. Postoperatively, tablet cifixime 200 mg twice daily, 0.025% xylomethazoline two drops in each nostril thrice daily, levocetrizine tablet 5 mg at bed time and ofloxacine 0.3% eye drop four times daily were prescribed for seven days.

Follow-up examinations were scheduled on the first and 14^th^ postoperative day, and after 1, 3, 6 and 12 months from the date of surgery. Subjective epiphora, if any, was evaluated with Munk′s score [Table 1].[4] Criteria for failure were non-patency on irrigation or tear meniscus height ≥2 mm in the postoperative period and most importantly, subjective epiphora beyond Munk’s score 1. Intranasal examinations with a nasal endoscope with video attachment were carried out for all the patients at two weeks and six months postoperatively. The operation time of each case was recorded.

**Table 1:** Munk’s score of epiphora[4]

*Grade*	
0	No epiphora
1	Occasional epiphora requiring dabbing less than twice a day
2	Epiphora requiring dabbing two to four times per day
3	Epiphora requiring dabbing 5-10 times per day
4	Epiphora requiring dabbing more than 10 times per day
5	Constant tearing

## Results

Ninety-six DCR surgeries were performed in this study. Females (62%) outnumbered males (35%). The age of the patients ranged from 21-69 years (mean age –41.0 ± 8.2 years). There was no significant intraoperative complication apart from excessive bleeding in one case. The average operation time was 45 minutes. One case was blocked on irrigation on first follow up day. Two cases had postoperative epiphora with Munk’s score 2 and 3, despite being patent on irrigation [Table 2]. The overall subjective satisfaction rate was 96.8%.

**Table 2:** Evaluation of results

	*Objective evidence (irrigation and tear meniscus height)*	*Subjective evidence epiphora (Munk's scale)*
Success	95	93
Failure	1	3
Percentage of success	98.9	96.8

Objective - Anatomical success; Subjective - Symptomatic success

The mean follow-up period was 13 months. Overall postoperative complications were five, out of which three patients had periorbital ecchymosis and remaining two patients complained of mild epistaxis [Table 3]. On intranasal examination by nasal endoscope, there was no stricture formation. On second follow-up day, one patient needed clot removal from osteotomy site via nasal endoscope.

**Table 3:** Postoperative complications

*Complication*	*No of patients*
Periorbital ecchymosis	3
Epistaxis (mild)	2

## Discussion

External dacryocystorhinostomy (DCR) is a highly successful procedure in managing epiphora due to nasolacrimal duct obstruction.[5,6] The reported success rate varies between 85% and 99%.[7] However, it is not technically easy and requires considerable experience as well as operative time. Meticulous attention to atraumatic handling of the soft tissues, a clear, properly placed and uniform rhinostomy with smooth edges, careful dissection to expose the true lumen of the lacrimal sac, followed by careful suturing of mucosal flaps, are important determinants of the outcome of the surgery. Moreover, individual response to tissue healing process is also an important factor for a successful DCR surgery.[8] In recent times, endonasal laser and intracanalicular laser DCR have been gaining in popularity over traditional DCR owing to advantages of no scar, less tissue damage and less intraoperative time. However, these procedures have their own limitations[8] and long term results are not yet available.

Traditional DCR has its own limitation with reported failure rates of 0 to 18%.[8-11] To overcome these, several authors have suggested different modifications of this technique from time to time.[3,12–14] We have presented a modification of the original technique by getting the benefit of double flap DCR through suturing both anterior and posterior flaps as well as the benefit of anterior suspended flap DCR by suspending the anterior flaps superiorly.

It is well proved that suturing both anterior and posterior mucosal flaps increases the probability of primary healing of the mucoal anastomosis and also reduces primary and secondary hemorrhage. [15,16] This results in a better postoperative outcome, as was reflected in our study. By suspending the anterior mucosal flaps, their adhesion with sutured posterior flaps can be avoided, which can contribute to failure of DCR. The sutures also close the deep planes of the wound. In this series, 96.8% cases had a postoperative epiphora of Munks scale 1 or less, which shows the effectiveness of this modification.

The role of intraoperative Mitomycin C application is debatable. Some studies has showed that intraoperative mitomycin C application increases the success rate of the surgical procedure[17,18] However, other studies have emphasized that mitomycin C does not improve the surgical outcome.[19,20]

To be adjudged a true success, assessment of both objective success (anatomical) and subjective success (symptomatic) of a procedure is important. In our series, two patients had persistent tearing despite anatomical success, while one patient had both anatomical and symptomatic failure. We believe that the modification of DCR described by us is simple and has the advantage of both double flap DCR and anterior suspension of anterior flaps, and is effective in the management of obstruction of lacrimal drainage system beyond the common canalicular opening. However, a larger comparative series would be more conclusive.

## References

[1] Toti A (1904). Nuovo metodo conservative di cura radicalle dellesupporazioni chronicle del sacco lacrimale. Clin Mod Firenze.

[2] Dupuy-Dutemps L, Boureguet J (1921). Procede plastique de dacryocystorhino stomy et ses resultants. Ann Ocul J.

[3] Baldeschi L, Nardi M, Hintschich CR, Koornnee L (1998). Anterior suspended flaps: A modified approach for external dacryocystorhinostomy. Br J Ophthalmol.

[4] Kuchar A, Stinkogler FJ (2001). Antegrade balloon dilatation of nasolacrimal duct obstruction in adults. Br J Ophthalmol.

[5] Tarbet KJ, Cluster PL (1995). External dacryocystorhinostomy: Surgical success, patient satisfaction and economic cost. Ophthalmoloy.

[6] Welham RA, Henderson PH (1973). Results of dacryocystorhinostomy: Analysis of causes for failure. Trans Ophthalmol Soc UK.

[7] Cokkeser Y, Everklioglu C (2000). Comparative external versus endoscopic dacryocystorhinostomy: Results in 115 patients. Otolaryngol Head Neck Surg.

[8] Deka A, Bhattachajee K, Bhuyan SK, Barua CK, Bhattacherjee H, Khaund G (2006). Effect of mitomycin C on ostium in dacryocystorhinostomy. Clin Exp Ophthalmol.

[9] You YA, Fang CT (2001). Intraoperative mitomycin C in dacryocystorhinostomy. Ophthal Plast Reconst Surg.

[10] Liao SL, Kao CS, Tseng JH, Chen MS, Hou PK (2000). Results of intraoperative mitomycin C application. Br J Ophthalmol.

[11] Walland MJ, Rose GE (1994). Factors affecting the success rate of open lacrimal surgery. Br J Ophthalmol.

[12] Ibrahim HA, Batterbury M, Banhegyi G, McGalliard J (2001). Endonasal laser dacryocystorhinostomy and external dacryocystorhinostomy outcome profile in a general ophthalmic service unit: A comparative retrospective study. Ophthalmic Surg Lasers.

[13] PicoG (1971). A modified technique of external dacryocystorhinostomy. Am J Ophthalmol.

[14] Iliff CE (1954). A simplified dacryocystorhinostomy. Tr Am Acad Ophthal.

[15] Jones BR (1773). Principles of lacrimal surgery. Trans Opthalmol Soc UK.

[16] Welham RAN, Wulc AE (1987). Management of unsuccessful lacrimal surgery. Br J Ophthalmol.

[17] Yildirim C, Yaylali V, Esme A, Ozden S (2007). Long-term results of adjunctive use of mitomycin C in external dacryocystorhinostomy. Int Ophthalmol.

[18] Liao SL, Kao CS, Tseng JH, Chen MS, Hou PK (2000). Results of intraoperative mitomycin C application. Br J Ophthalmol.

[19] Liu D, Bosley TM (2003). Silicone nasolacrimal intubation with mitomycin C. A prospective, randomized, doublemasked study. Ophthalmology.

[20] Roozitalab MH, Amirahmadi M, Namazi MR (2004). Results of the application of intraoperative Mitomycin. Eur J Ophhalmol.

